# ‘To improve is to change’—but is it safe? A single surgeon’s transition from lateral to vertical hemispherotomy

**DOI:** 10.1007/s00381-026-07353-2

**Published:** 2026-06-19

**Authors:** Omar Salim, Aswin Chari, M. Zubair Tahir

**Affiliations:** 1 Department of Clinical Neurosciences, Royal Infirmary of Edinburgh, Edinburgh, UK; 2 Department of Neurosurgery, Great Ormond Street Hospital, London, UK; 3 Developmental Neurosciences, Great Ormond Street Institute of Child Health, University College London, 30 Guilford Street, WC1N 3EH, London, UK

**Keywords:** Epilepsy surgery, Hemispherotomy, Paediatric, Drug-resistance

## Abstract

**Purpose:**

Hemispherotomy is an established treatment for children with drug-resistant epilepsy. Recent reports have suggested a superiority of outcomes with the vertical approach compared to the traditional lateral approach. This study investigated whether an experienced epilepsy surgeon can safely transition from lateral peri-Sylvian to vertical parasagittal hemispherotomy, through evaluation of postoperative seizure freedom and associated outcomes.

**Methods:**

Thirty-six patients who underwent hemispherotomy by a single surgeon between 2016 and 2025 were analysed, 23 lateral peri-Sylvian and 13 vertical parasagittal. Pre-, peri- and postoperative findings were compared across the two techniques, with Pearson’s chi-squared, Fisher’s exact and Mann-Whitney *U* tests utilised where appropriate to detect significant differences. Patients with 1-year postoperative follow-up were split into chronological epochs. A binary logistic regression model compared seizure freedom by epoch whilst controlling for factors shown to predict seizure freedom.

**Results:**

90.9% of the lateral group and 83.3% of the vertical group were seizure free (Engel class I) at 1-year postoperative follow-up (*p* = 0.602), with no statistically significant differences in the complication rates. Mean operative duration was significantly less for the vertical compared to the lateral group (340.9 vs 448.8 min, *p* = 0.002), as was intraoperative haemoglobin drop (18.1 vs 27.2 g/L, *p* = 0.002). Mean hospital stay was comparable (8.7 vs 9.8 days, *p* = 0.871). There was no statistically significant difference in 1-year seizure freedom rate noted by chronological epoch whilst adjusting for factors predicting seizure freedom (*p* = 0.816).

**Conclusion:**

This study demonstrates that a single experienced epilepsy surgeon can safely transition between techniques, without compromising on seizure freedom outcomes or incurring additional morbidity. Whilst larger studies may demonstrate the favourability of one technique over the other, it may be useful to the individual surgeon to have a sound grasp of both techniques in their armamentarium.

## Introduction

Approximately one in three children with epilepsy will become refractory to antiseizure medications (ASMs) [1]. Such drug-resistant epilepsy (DRE) increases the risk of cognitive and developmental regression, poorer quality of life and early mortality [2, 3]. Some DRE cases are associated with hemispheric abnormalities, which may arise from various aetiologies spanning congenital lesions (e.g. diffuse cortical dysplasia, hemimegalencephaly), progressive hemispheric disorders (e.g. Sturge-Weber syndrome, Rasmussen’s encephalitis) and acquired diffuse hemispheric insults (e.g. perinatal stroke or infection) [4, 5].

**Fig. 1:**
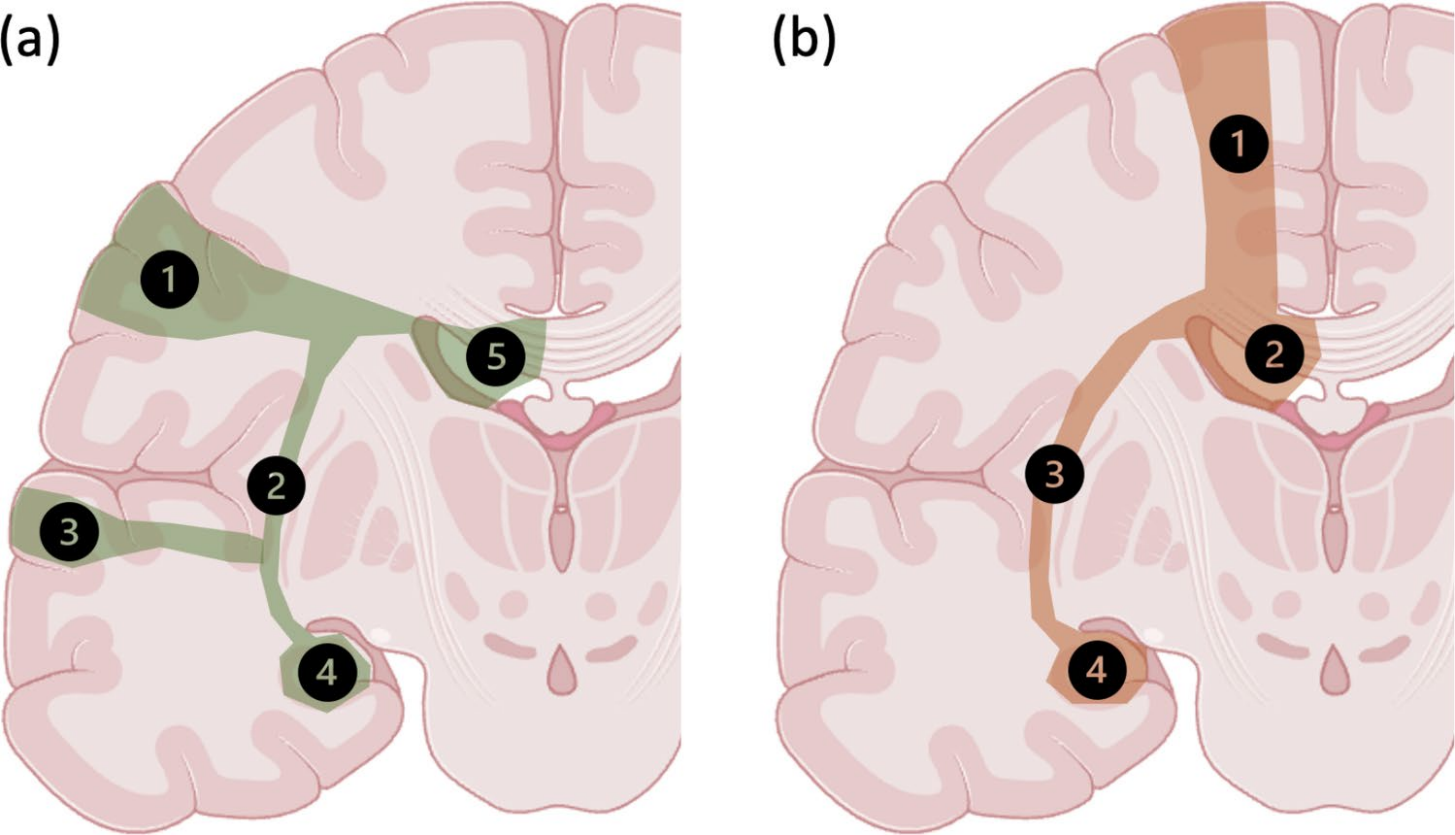
Diagrammatic representation of **a** lateral peri-Sylvian and **b** vertical parasagittal hemispherotomy on coronal brain diagram. **a** Lateral peri-Sylvian hemispherotomy showing (1) supra-Sylvian disconnection, (2) insular undercut, (3) infra-Sylvian disconnection, (4) temporal disconnection and (5) corpus callosotomy. **b** Vertical parasagittal hemispherotomy showing (1) the superior frontal gyrus window to enter the ventricle, (2) corpus callosotomy, (3) insular undercut and (4) temporal disconnection

Surgery to anatomically resect (hemispherectomy) or functionally isolate (hemispherotomy) the affected cerebral hemisphere is an established treatment [6]. The first case report of anatomical hemispherectomy to control seizures was described in 1938 by McKenzie [7], with the first case series by Krynauw in 1950 [8] and a functional disconnection method later proposed by Rasmussen [9]. The approach has since undergone various modifications and iterations. Modern practice focuses largely on hemispherotomy, involving transection of the commissural fibres and projections of a complete hemisphere, whilst minimising cortical resection [6, 10]. Currently, hemispherotomy surgeries employ either the widely-practiced and established lateral approach (via a peri- or trans-Sylvian route) [6, 10–12] or the more recent vertical parasagittal method (Fig. 1) [13]. The goal of such surgeries is not only seizure cessation or reduction, but also slowing neurocognitive and behavioural decline [11, 14].

In appropriately selected patients, hemispherotomy displays good rates of seizure freedom (around 60–90% [15–22]) and low complication rates, aside from anticipated neurological sequelae (e.g. hemiparesis, homonymous hemianopia) [4, 5, 17]. The Hemispherectomy Outcome Prediction Scale (HOPS) study was a large, multi-institution study which identified five independent predictive variables significantly associated with seizure freedom post-hemispheric surgery [4]. These comprised age older than 3.5 years at seizure commencement, stroke as the underlying epilepsy aetiology, absence of previous resective surgery, absence of generalised seizure symptomatology and absence of contralateral hypometabolism on 18 fluorodeoxyglucose-positron emission tomography (FDG-PET) [4]. More recently, these findings were combined with three further variables (lesion presence on magnetic resonance imaging (MRI), age at surgery and history of infantile spasms) to devise a HOPS score that predicts likelihood of seizure freedom at different timepoints following hemispheric surgery [23]. Comparable findings were noted in a large systematic review and meta-analysis, with the additional finding of absence of contralateral interictal epileptic activity on electroencephalography (EEG) as a significant predictor of seizure freedom [24].

Regarding choice of hemispherotomy technique, Fallah et al. [16] undertook a post hoc analysis of the original HOPS study and found the vertical method produced significantly better long-term postoperative seizure freedom rates than the lateral approach. From a morbidity perspective, the vertical approach may also be preferred as it requires less cortical resection and time to perform, with fewer associated blood transfusions and complications [25]. The technique chosen by an individual surgeon is largely determined by their training and exposure to each approach. It is not known whether peri- and postoperative outcomes will initially be affected as the alternative vertical approach is learned, or whether one can safely transition between techniques.

There is perhaps an emerging impetus for established surgeons using the lateral approach to adopt the vertical approach, with aims of either optimising outcomes or having both techniques in their surgical armamentarium for selected cases. The aim of this study was to determine whether an experienced epilepsy surgeon can safely transition from a lateral to a vertical hemispherotomy approach. This is evaluated through comparison of seizure freedom rates, whilst accounting for factors highlighted by the HOPS study as predicting seizure outcome.

## Methods

This was a retrospective single-centre, single-surgeon cohort study of prospectively collected data. It is reported according to the STROBE guidelines. It was approved as a service evaluation at our institution (ID: 4468), and individual patient consent was waived.

The medical records for all consecutive patients who underwent first hemispherotomy by the same epilepsy surgeon (ZT) between 2016 and 2025 at a single tertiary referral paediatric neurosurgery centre were analysed. All cases underwent thorough presurgical evaluation, and the procedure was offered following discussion in the epilepsy surgery multidisciplinary team meeting.

The senior author is an experienced, fellowship-trained epilepsy surgeon well-versed in the interhemispheric approach for corpus callostomy and the trans-cortical approach for frontal lobe disconnection and resection of subependymal giant cell astrocytoma. He was trained in the peri-Sylvian procedure in accordance with the method proposed by Villemure et al. [10]. Following significant experience with this technique, a decision was made in 2022 to switch to the vertical parasagittal technique, as described by Delalande et al. [13], with the aim of improving seizure and morbidity outcomes for patients and expanding the senior author’s surgical repertoire. The transition was facilitated by significant surgical experience and a sound understanding of grey and white matter anatomy and its relationship with the ventricles. This background experience was refined prior to undertaking the vertical hemispherotomy technique through reading published literature on different approaches, observation of surgical videos and studying detailed white matter anatomy. Additional knowledge was gained using augmented reality and brain models to appreciate the steps involved in vertical disconnection.

Data was collected across the following parameters: sex, age, seizure semiology, aetiology, preoperative symptoms, ASMs trialled, EEG findings, MRI findings, HOPS score, operative details, postoperative complications, seizure outcomes (in accordance with the Engel Epilepsy Surgery Outcome Scale) [26], residual connections on postoperative MRI and subdural cerebrospinal fluid (CSF) on postoperative MRI. For patients who underwent re-do hemispherotomy, seizure outcomes were recorded until the last follow-up appointment before the second surgery. HOPS scores were calculated using the online calculator at https://hops-calculator.com/, and the probability of seizure freedom at 12 months postoperatively was noted. Short focal seizures not affecting conscious level and those occurring within the first week following surgery were not regarded as events, as in Weil et al. [4].

Demographic and patient history data was summarised using descriptive statistics: mean, range and standard deviation for continuous variables and frequency for categorical variables. The two surgical groups (vertical and lateral hemispherotomies) were compared for significant differences via the Pearson’s chi-squared or Fisher’s exact tests (when over 20% of expected counts were less than 5) for dichotomous variables, as appropriate. Continuous variables were compared between the groups using the Mann-Whitney *U* test. Patients with at least 1 year of postoperative follow-up were then split into 3 chronological epochs. A binary logistic regression model was run comparing 1-year seizure freedom (Engel class I) between epochs whilst adjusting for HOPS score. This regression was performed to determine whether a significant learning curve was incurred with time (and transition from lateral to vertical techniques) with regard to seizure freedom at 12 months, controlling for those factors determined in the HOPS study to significantly impact seizure freedom postoperatively. Statistical significance was regarded as *p* < 0.05 for all tests. Statistical analyses were performed in SPSS Statistics version 29 (IBM, Armonk, NY, USA).

The dataset is available from the corresponding author on reasonable request.

## Results

Thirty-six (36) hemispherotomy procedures were identified; the first 23 through a lateral approach and the next 13 via the vertical approach.

### Demographic and clinical findings

Preoperative demographic and patient history data are summarised in Table 1.

**Table 1:** Preoperative demographic and patient history data of the study cohort

Preoperative characteristic		Prevalence: ***N***/cohort total or ***N***/subgroup total (%) or ***N*** (± SD, range)			
		Total	Vertical	Lateral	Test for significant difference
Sex	Male	16/36 (44.4%)	5/13 (38.5%)	11/23 (47.8%)	*p* = 0.587
	Female	20/36 (55.6%)	8/13 (61.5%)	12/23 (52.2%)	
Age at seizure onset (mean)		2.3 years (± 2.5, 0.003–7.8)	2.2 years (± 2.3, 0.003–4.8)	2.4 years (± 2.7, 0.003–7.8)	*p *= 0.897
Age at surgery (mean)		8.7 years (± 5.2, 0.7–17.6)	11.1 years (± 5.1, 1.6–17.6)	7.3 years (± 4.8, 0.7–17.3)	*p* = 0.040*
Duration of preoperative epilepsy		6.4 (± 4.4, 0.3–15.8) years	9.1 years (± 4.7, 1.2–15.8)	4.9 years (± 3.5, 0.3–11.2)	*p* = 0.016*
Epilepsy aetiology	Porencephalic cyst/stroke	24/36 (66.7%)	9/13 (69.2%)	15/23 (65.2%)	*p* = 0.888
	Rasmussen’s encephalitis	7/36 (19.4%)	2/13 (15.4%)	5/23 (21.7%)	
	Sturge-Weber syndrome	3/36 (8.3%)	1/13 (7.7%)	2/23 (8.7%)	
	Hemimegalencephaly	1/36 (2.8%)	1/13 (7.7%)	0/23 (0%)	
	Malformation of cortical development	1/36 (2.8%)	0/13 (0%)	1/23 (4.3%)	
Seizure type	Generalised	33/36 (91.7%)	13/13 (100%)	20/23 (87.0%)	*p* = 0.288
	Non-generalised**	3/36 (8.3%)	0/13 (0%)	3/23 (13.0%)	
	Focal**	34/36 (94.4%)	12/13 (92.3%)	22/23 (95.7%)	*p* = 1.000
	Tonic-clonic	24/36 (66.7%)	11/13 (84.6%)	13/23 (56.5%)	*p* = 0.143
	Absence/vacant/staring spells	15/36 (41.7%)	7/13 (53.8%)	8/23 (34.8%)	*p* = 0.265
	Infantile spasms	13/36 (36.1%)	3/13 (23.1%)	10/23 (43.5%)	*p* = 0.292
	Drop attacks/head drops/atonic	11/36 (30.6%)	3/13 (23.1%)	8/23 (34.8%)	*p* = 0.708
	Status epilepticus	5/36 (13.9%)	2/13 (15.4%)	3/23 (13.0%)	*p* = 1.000
	Myoclonic jerks	3/36 (8.3%)	0/13 (0%)	3/23 (13.0%)	*p* = 0.288
Multiple seizures per day	Yes	33/36 (91.7%)	12/13 (92.3%)	21/23 (91.3%)	*p* = 1.000
	No	3/36 (8.3%)	1/13 (7.7%)	2/23 (8.7%)	
Preoperative neurological symptoms	Hemiplegia/hemiparesis	36/36 (100%)			
	Homonymous hemianopia/visual field defect	12/36 (33.3%)	2/13 (15.4%)	10/23 (43.5%)	*p* = 0.143
	Intellectual disability	12/36 (33.3%)	6/13 (46.2%)	6/23 (26.1%)	*p* = 0.281
	Cerebral palsy	10/36 (27.8%)	3/13 (23.1%)	7/23 (30.4%)	*p* = 0.716
	Neglect/semi-neglect	4/36 (19.4%)	3/13 (23.1%)	1/23 (4.3%)	*p* = 0.124
	Spasticity, hyperreflexia	4/36 (11.1%)	2/13 (15.4%)	2/23 (8.7%)	*p* = 0.609
	Dystonia/hemi-dystonia	4/36 (11.1%)	2/13 (15.4%)	2/23 (8.7%)	*p* = 0.609
	Tremor	1/36 (2.8%)	1/13 (7.7%)	0/23 (0%)	*p* = 0.361
	Headache	1/36 (2.8%)	0/13 (0%)	1/23 (4.3%)	*p* = 1.000
ASMs at surgery*** (mean)		3.0 (± 1.0, 1–6)	2.8 (± 1.2, 1–5)	3.2 (± 0.9, 2–6)	*p* = 0.344
Total ASMs trialled preoperatively*** (mean)		5.6 (± 1.8, 2–9)	5.6 (± 2.0, 2–9)	5.6 (± 1.7, 2–9)	*p* = 0.974
Previous surgery	Yes	7/36 (19.4%)	2/13 (15.4%)	5/23 (21.7%)	*p* = 1.000
	No	29/36 (80.6%)	11/13 (84.6%)	18/23 (78.3%)	
Preoperative interictal EEG	Ipsilateral	19/35 (54.3%)	7/13 (53.8%)	12/22 (54.5%)	*p* = 0.968
	Bilateral/non-lateralising	16/35 (45.7%)	6/13 (46.2%)	9/22 (45.5%)	
Preoperative ictal EEG	Ipsilateral	24/32 (75.0%)	8/9 (88.9%)	16/23 (69.6%)	*p* = 0.386
	Bilateral/non-lateralising	8/32 (25.0%)	1/9 (11.1%)	7/23 (30.4%)	
Hemispheric lesion on MRI	Ipsilateral	35/36 (97.2%)	13/13 (100%)	22/23 (95.7%)	*p* = 1.000
	Bilateral	1/36 (2.8%)	0/13 (0%)	1/23 (4.3%)	

*N* = 36. Data is presented as total prevalence and divided by vertical and lateral hemispherotomy subgroup. Since patients often displayed more than one seizure type and preoperative neurological symptom, statistical analyses were run for each individual category within these characteristics

*N* Number of patients for categorical variables or mean value for continuous variables, *SD* Standard deviation, *ASM* Antiseizure medication

*Statistically significant difference (*p* < 0.05)

**Non-generalised seizures comprised those patients only displaying focal seizures. Focal seizure type includes focal with preserved consciousness, focal with impaired consciousness, focal motor, focal tonic, focal clonic, auras, gelastic, dyscognitive, complex partial and epilepsy partialis continua (EPC)

***Includes steroids

The two groups were comparable in terms of preoperative characteristics. The vertical group was significantly older at surgery (11.1 vs 7.3 years, *p* = 0.040), with a longer epilepsy duration before surgery (9.1 vs 4.9 years, *p* = 0.016). The most common underlying epilepsy aetiology was porencephalic cyst/stroke, with no significant difference comparing aetiology by hemispherotomy approach (*p* = 0.888). There were no statistically significant differences in preoperative seizure types or neurological deficits between groups.

Seven patients had undergone surgery previously. The vertical group comprised one patient who previously underwent a left frontal disconnection with later vagal nerve stimulator (VNS) insertion and a second who had undergone left frontal lobectomy. The lateral group comprised one patient who had Gamma knife surgery for a ruptured AVM, two ventriculoperitoneal (VP) shunt insertions (for congenital and post-meningitic hydrocephalus, the latter of whom also had a VNS insertion), and two patients who underwent biopsies.

There were no differences in preoperative ictal or interictal EEG lateralisation. The vast majority of patients had ipsilateral lesions on MRI (97.2%), with one lateral group patient displaying bilateral lesions. This patient with porencephalic cystic encephalomalacia was shown to have a contralateral basal ganglia lesion on imaging.

### Operative findings and postoperative symptoms

Table 2 summarises the associated operative findings and postoperative symptoms.

**Table 2:** Operative and postoperative findings of the cohort

Operative/postoperative findings		Prevalence: *N*/cohort total or *N*/subgroup total (%) or *N* (± SD, range)			
		Total	Vertical	Lateral	Test for significant difference
Hemispherotomy type		36	13/36 (36.1%)	23/36 (63.9%)	
Hemispherotomy side	Left	19/36 (52.8%)	7/13 (53.8%)	12/23 (52.2%)	*p* = 0.923
	Right	17/36 (47.2%)	6/13 (46.2%)	11/23 (47.8%)	
Operative duration (mean)		394.8 min (± 91.2, 277–585)	340.9 min (± 56.4, 277–488)	448.8 min (± 88.5, 312–585)	*p* = 0.002*
Intraoperative complications		1/36 (2.8%)	0/13 (0%)	1/23 (4.3%)	*p* = 1.000
Haemoglobin drop (mean)		23.9 g/L (± 10.0, 3–58)	18.1 g/L (± 6.8, 3–29)	27.2 g/L (± 10.0, 6–58)	*p* = 0.002*
Blood transfusions		6/36 (16.7%)	0/13 (0%)	6/23 (26.1%)	*p* = 0.068
Acute postoperative seizures		6/36 (16.7%)	2/13 (15.4%)	4/23 (17.4%)	*p* = 0.877
EVD/VP shunt inserted		1/36 (2.8%)	0/13 (0%)	1/23 (4.3%)	*p* = 0.581
Postoperative complications		4/36 (11.1%)	1/13 (7.7%)	3/23 (13.0%)	*p* = 1.000
Total hospital stay (mean)		9.4 days (± 5.1, 4–27)	8.7 days (± 2.8, 5–13)	9.8 days (± 6.0, 4–27)	*p* = 0.871
Follow-up duration (mean)		2.9 years (± 2.1, 0.2–8.3)	1.5 years (± 0.7, 0.9–3.1)	3.6 years (± 2.3, 0.2–8.3)	*p* = 0.002*

*N* = 36. Operative duration data available for 26 patients (13 vertical, 13 lateral). Haemoglobin drop calculated from preoperative full blood count and day 1 postoperative full blood count (or lowest intraoperative arterial blood gas haemoglobin if not available or transfused intraoperatively). Acute postoperative seizures = those occurring within the first week post-surgery. Subgroup refers to either the vertical or lateral hemispherotomy patient group

*N* Number of patients for categorical variables or mean value for continuous variables, *SD* Standard deviation

*Statistically significant difference (*p* < 0.05)

Mean operative duration was significantly shorter for the vertical compared to the lateral hemispherotomy subgroup (340.9 vs 448.8 min; *p* = 0.002). Intraoperative haemoglobin drop was also significantly lower (18.1 vs 27.2 g/L; *p* = 0.002). Six patients required blood transfusion, all in the lateral group, although this did not reach statistical significance (*p* = 0.068).

Intraoperative complications were rare, noted in only one lateral group patient. This involved folding of the thin cortical mantle during the operation with subsequent venous bleeding from bridging veins; a decision was made to not perform the corpus callostomy to avoid further venous bleeding. Acute postoperative seizures occurred in 2 vertical patients (15.4%) and 4 lateral patients (17.4%, *p* = 0.877). Only one lateral group patient underwent planned temporary external ventricular drain (EVD) placement due to ventriculomegaly on preoperative MRI (*p* = 0.581).

**Fig. 2:**
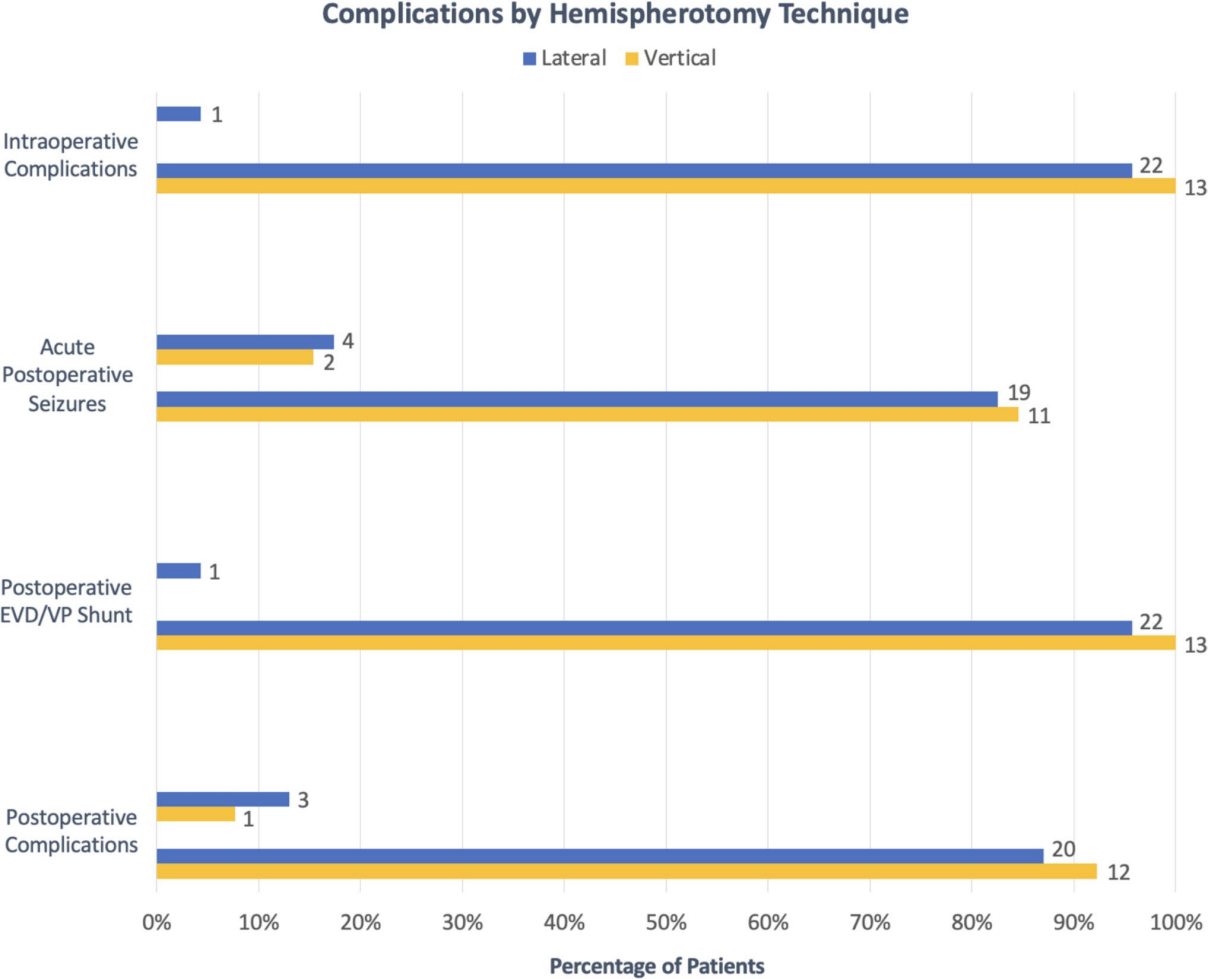
Summary of complications observed by hemispherotomy technique (vertical parasagittal vs lateral peri-Sylvian). Group sizes: lateral hemispherotomy (*n* = 23), vertical hemispherotomy (*n* = 13), total *N* = 36. Bars depict the percentage of complications by hemispherotomy subgroup. Numbers adjacent to bars represent patient counts. Acute postoperative seizures are those occurring within the first week following surgery. EVD, external ventricular drain; VP, ventriculoperitoneal

There were no instances of wound infection, cerebral infarction or mortality. The single intraoperative complication, from the lateral group, comprised an elective decision to not perform corpus callostomy due to cortical folding of the thin mantle and risk of venous bleeding. Four patients developed postoperative complications (one vertical, three lateral; *p* = 1.00) (Fig. 2). Following vertical hemispherotomy, one patient experienced a deterioration in expressive language. This patient had surgery on the left (dominant) hemisphere; hence, the deficit is consistent with anticipated neuroanatomical outcomes. The lateral group complications comprised one third nerve palsy and two patients with persistent postoperative pyrexia without wound problems requiring intravenous antibiotics.

Average hospital stay was comparable between groups (8.7 vs 9.8 days, *p* = 0.871). Due to the more recent nature of vertical surgeries, mean follow-up duration was significantly shorter for this subgroup (1.5 vs 3.6 years, *p* = 0.002). At most recent follow-up, all patients displayed anticipated contralateral hemiplegia/hemiparesis and hemi-visual field defects. Two patients (one vertical, one lateral) experienced transient headaches which resolved with time.

### Postoperative imaging, seizure findings and learning curve

Imaging findings and seizure outcomes are summarised in Table 3. Residual white matter connections were observed in two patients, both in the lateral group, with no significant difference between surgical approaches (*p* = 0.519). One vertical patient was referred for VNS surgery and two lateral patients underwent re-do surgery. The re-do hemispherotomies involved patients with seizure recurrence 12 months and 3 years following their first surgeries. The former patient showed no obvious residual connection, but additional frontal disconnection was carried out close to the Sylvian fissure. The latter had residual connections in the fronto-basal and post-insular regions, with peri-Sylvian disconnection performed.

**Table 3:** Postoperative imaging and seizure outcome findings across the cohort

Postoperative imaging/seizure outcome	Prevalence: *N*/cohort total or *N*/subgroup total (%) or % (± SD, range)			
	Total	Vertical	Lateral	Test for significant difference
Subdural CSF collection on postoperative MRI	9/35 (21.9%)	4/13 (30.8%)	5/22 (22.7%)	*p* = 0.698
Residual white matter connection on postoperative MRI	2/35 (5.7%)	0/13 (0%)	2/22 (9.1%)	*p* = 0.519
Re-do surgery	3/35 (10.7%)	1/13 (7.7%)	2/22 (9.1%)	*p* = 1.000
Seizure freedom (Engel class I) at last follow-up	28/36 (77.7%)	11/13 (84.6%)	17/23 (73.9%)	*p* = 0.682
Seizure freedom (Engel class I) at 1-year follow-up	30/34 (88.2%)	10/12 (83.3%)	20/22 (90.9%)	*p* = 0.602
HOPS probability of seizure freedom (Engel class I) at 1 year (mean)	85.0% (± 7.5, 52.8–94.2)	85.7% (± 6.3, 72.1–92.9)	84.5% (± 8.1, 52.8–94.2)	*p* = 0.434

*N* = 36. One lateral group patient was lost to follow-up after approximately 2 months, hence the lower total patients within associated categories. In addition, one vertical patient fell just short of the 1-year follow-up mark, as they were reviewed in clinic at 11 months postoperatively

*N* Number of patients for categorical variables or mean value for continuous variables, *SD* Standard deviation, *HOPS* Hemispherectomy outcome prediction scale

At last follow-up, 28 patients (77.7%) were seizure free (Engel I). Among the eight who were not, five were Engel II (13.9%), two Engel III (5.6%) and one Engel IV (2.8%). By subgroup, 11/13 vertical patients (84.6%) and 17/23 lateral patients (73.9%) were seizure free. Among the two vertical group patients (15.4%) who were not, one was Engel II (7.7%) and the other Engel III (7.7%). The six lateral group patients who were not comprised four Engel II (17.4%), one Engel III (4.3%) and one Engel IV (4.3%).

At 1 year, 10/12 vertical patients (83.3%) and 20/22 lateral patients (90.9%) were seizure free (Engel I; *p* = 0.602). These outcomes were comparable to the predicted probability of seizure freedom (Engel I) using HOPS (85.7 ± 6.3% for vertical and 84.5 ± 8.1% for lateral hemispherotomies).

**Fig. 3:**
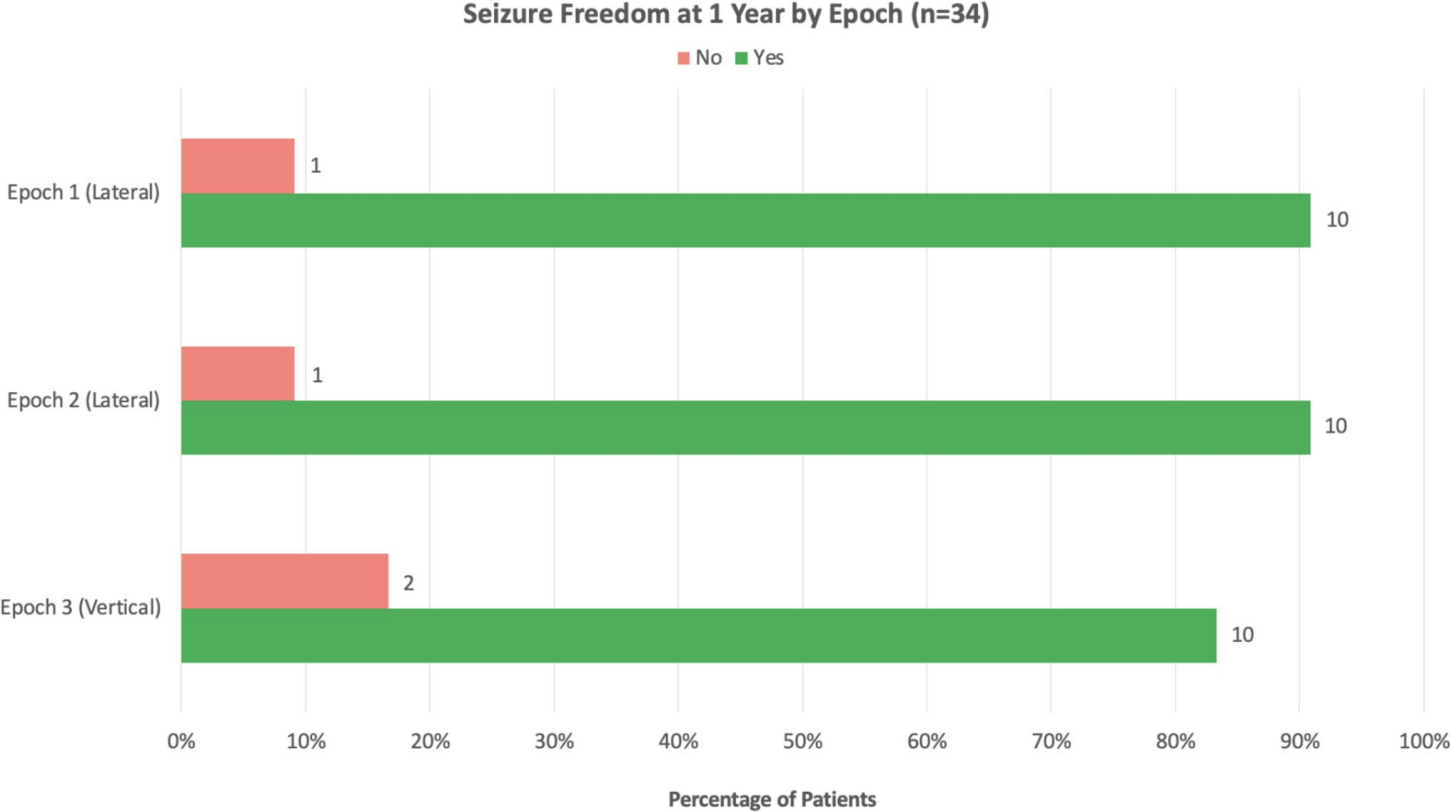
Seizure freedom rate for those patients followed up at 1 year postoperatively. Group sizes: epoch 1 (*n* = 11), epoch 2 (*n* = 11), epoch 3 (*n *= 12), total *N* = 34. Two patients were excluded due to insufficient follow-up duration. Bars depict the percentage of patients achieving seizure freedom at 1 year postoperatively by epoch. Numbers adjacent to bars represent patient counts. No significant difference in seizure freedom was noted across chronological epoch after adjusting for HOPS prediction (*p* = 0.816)

The 34 patients with 1-year follow-up were split into 3 chronological epochs comprising 2 lateral hemispherotomy groups (*n* = 11 each) and one vertical hemispherotomy group (*n* = 12). On binary logistic regression, there was no significant difference in 1-year seizure freedom (Engel I) between epochs after adjusting for HOPS prediction of seizure freedom (*p* = 0.816) (Fig. 3).

## Discussion

This study explores the experiences of a single epilepsy surgeon transitioning from a lateral peri-Sylvian hemispherotomy approach to a vertical method to treat DRE in a cohort of paediatric patients. It demonstrates the safety of this transition without a compromise in postoperative seizure outcomes or complications.

The incidence of seizure freedom at last follow-up was greater for the vertical hemispherotomy group (84.6%) compared to the lateral group (73.9%), although this difference was not significant (*p* = 0.682). It should be noted, however, that the lateral group was followed up for a significantly longer mean duration (3.6 vs 1.5 years, *p* = 0.002). Similarly, although higher seizure freedom at last follow-up was noted for vertical (84.2%) over lateral (73.1%) techniques in de Palma et al. [18], this difference was not significant. Fallah et al. [16] also reported a greater seizure freedom rate at last follow-up for vertical hemispherotomy over lateral (86.1% vs 79.0%), although again the difference was not significant. A much longer follow-up duration (6 years and above) may be warranted to elicit any differences in durable seizure freedom between the techniques [16].

Operative duration and intraoperative haemoglobin drop were significantly lower for the vertical compared to the lateral hemispherotomy subgroups (both *p* = 0.002). As highlighted in Iwasaki et al. [25], these parameters are a useful measure of comparison due to the reduction in cortical resection that occurs using the vertical approach, with associated implications for lower morbidity and faster recovery postoperatively.

Determining whether the transition from lateral to vertical hemispherotomy approaches incurred a learning curve in this cohort involved comparing seizure freedom at 1 year by chronological epoch whilst controlling for HOPS score. The latter was chosen due to its ease of use as a summary measure for variables shown to significantly predict seizure freedom in a large, recent multi-centre study [23]. No significant difference was noted between epochs. This shows strong baseline outcomes, with no change between epochs 1 and 2 as the surgeon gained procedural experience. The lack of change between epochs 2 and 3 also reflects no deleterious impact on seizure outcomes during the transition from lateral to vertical. Nevertheless, this reflects a single surgeon practicing in a busy tertiary referral centre. More data from other surgeons and centres will be needed to generalise these findings. As highlighted in Fallah et al. [16], longer durations of follow-up will also be warranted to determine whether switching approach results in sustained improvements in postoperative outcomes and seizure freedom. Indeed, it may be the case that certain cases are more suited to a particular technique and, rather than a one-size-fits-all approach, surgeons may select a method based on the individual patient; it may thus be useful to have both techniques available.

For an epileptic aetiology like hemimegalencephaly, the interhemispheric approach of vertical hemispherotomy is potentially more preferable due to marked hemispheric enlargement and midline shift, as well as distortion and poor development of the ventricles. This may also be the case for malformations of cortical development, whereby abnormalities in gyral and ventricular anatomy may favour the clearer identification of anatomical landmarks afforded by the vertical technique. Conversely, lateral hemispherotomy may be less technically challenging in cases of acquired lesions like perinatal strokes and porencephalic cysts with largely preserved ventricular structure. Similarly, for epilepsy secondary to Rasmussen’s encephalitis and Sturge-Weber syndrome, hemispheric anatomy is generally maintained. This means either hemispherotomy technique may be employed; however, the lateral method may facilitate a more direct approach using established cortical landmarks. Nevertheless, whilst proficiency in both techniques is desirable, in clinical practice, many tertiary centres carry out only a small number of such surgeries each year, potentially limiting the capacity to acquire and maintain expertise in both approaches.

There exist some limitations inherent to the study design. The sample size was small, limiting robust statistical comparisons between the techniques in terms of safety and efficacy. This also reduces the sensitivity of the binary logistic regression to identify meaningful differences between the chronological epochs. The majority of the cohort had porencephalic cyst or stroke as the underlying seizure aetiology, with a typically well-defined epileptogenic zone and less complexity than developmental pathologies. For such pathologies, either hemispherotomy approach is likely to achieve adequate disconnection. This may account for the absence of a significant difference in seizure outcome between approaches, since any differences between techniques are less readily detectable—for example, the potential advantage of more complete and less technically challenging insular disconnection through vertical hemispherotomy. By contrast, work in the medical literature demonstrating seizure outcome differences between the two hemispherotomy techniques has involved more heterogenous aetiologies, whereby anatomical complexity of the lesions may place greater emphasis on extent of disconnection [16]. The significantly longer follow-up duration for the lateral over the vertical group restricts any meaningful comparisons beyond the 1-year postoperative mark. Some useful parameters for comparing the two surgical groups were not collated in this study due to missing data. These included functional FDG-PET data, as only three patients had this investigation documented in their preoperative work up. Whilst this investigation is often not conducted in patients with a clear epileptogenic lesion on MRI, the multi-centre HOPS study identified its significant association with seizure freedom following hemispheric surgery. When comparing seizure outcome by chronological epoch, the HOPS score was used to control for factors influencing seizure freedom. Whilst this scoring system uses specific variables identified in the large, multi-centre HOPS study as significantly correlated with seizure freedom, other variables may have been excluded which also influence seizure semiology prognosis.

## Conclusion

This study shows that a single epilepsy surgeon can safely transition from a lateral peri-Sylvian hemispherotomy approach to a vertical approach without impacting upon seizure freedom rates or complications. Whilst it may be beneficial to assess whether this applies to a wider range of surgeons, it may be possible that mastering both approaches may have its benefits, giving a surgeon the option to tailor their approach to optimise outcomes for specific patients.

## Data Availability

The dataset supporting the study findings is available from the corresponding author upon reasonable request.

## Declarations

### Conference presentations

Data partly presented as a poster presentation at the International Society of Pediatric Neurosurgery 2024 Meeting, Toronto, Canada, October 2024.
